# Effect of Crosslinking Agents on Chitosan Hydrogel Carriers for Drug Loading and Release for Targeted Drug Delivery

**DOI:** 10.3390/gels10070421

**Published:** 2024-06-26

**Authors:** Md Salah Uddin, Suyash Khand, Chao Dong

**Affiliations:** 1 Department of Mechanical Engineering, University of Texas Permian Basin, Odessa, TX 79762, USA; khand_s53116@utpb.edu; 2 Department of Chemistry, University of Texas Permian Basin, Odessa, TX 79762, USA; dong_c@utpb.edu

**Keywords:** targeted drug delivery, chitosan, hydrogel, crosslinking agent, cancer

## Abstract

Numerous studies report on chitosan hydrogels in different forms, such as films, porous structures, nanoparticles, and microspheres, for biomedical applications; however, this study concentrates on their modifications with different crosslinking agents and observes their effects on drug loading and releasing capacities. Linear chitosan, along with chitosans crosslinked with two major crosslinkers, i.e., genipin and disulfide, are used to formulate three different hydrogel systems. The crosslinking process is heavily impacted by temperature and pH conditions. Three different drugs, i.e., thymoquinone, gefitinib, and erlotinib, are loaded to the hydrogels in de-ionized water solutions and released in phosphate-buffered solutions; thus, a total of nine combinations are studied and analyzed for their drug loading and releasing capabilities with ultraviolet–visible (UV–Vis) spectroscopy. This study finds that thymoquinone shows the lowest loading efficacy compared to the two other drugs in all three systems. Gefitinib shows stable loading and releasing regardless of crosslinking system, and the genipin-crosslinked system shows stable loading and releasing with all three drug molecules. These experimental results agree well with the findings of our previously published results conducted with molecular dynamics simulations.

## 1. Introduction

Drug delivery is the process of administering medication to the body to achieve a therapeutic effect. Drug delivery is an important field of study in medicine and pharmaceuticals, and ongoing research is focused on developing new drug delivery systems and techniques to improve drug delivery outcomes and patient care. Targeted drug delivery refers to the administration of therapeutic agents or drugs to targeted sites within the body for a specific treatment to maximize the therapeutic benefit of drugs while minimizing their toxic side effects [1,2]. Drug delivery systems are designed to improve the pharmacokinetics (movement of drugs) and pharmacodynamics (biological response of drugs and the corresponding mechanism) of drugs by controlling the rate, duration, and distribution of drug release [3]. Drug delivery systems can be classified into several categories based on their modes of action. Controlled-release systems release drugs over an extended period, which improves patient compliance and reduces the frequency of drug administration. The drug delivery systems implanted into the body release drugs over a long period at a sustained rate to areas such as the brain or spinal cord minimizing the need for repeated drug administration. Inhalation systems deliver drugs to the lungs for the treatment of respiratory diseases. Transdermal systems deliver drugs through the skin for the treatment of skin diseases or systemic drug delivery. Drugs are taken by mouth in the form of pills, capsules, or liquids in oral drug delivery. Topical drug delivery systems are applied to the skin or mucous membranes, such as in the form of creams, ointments, gels, or patches. Drugs are delivered to the lungs through inhalers, nebulizers, or other devices for inhalation drug delivery. Drugs are delivered directly into the body through injections, including intravenous (into a vein), intramuscular (into a muscle), and subcutaneous (under the skin) injections, with injectable drug delivery. Suppository drug delivery inserts drugs into the rectum or vagina in the form of suppositories. Targeted drug delivery is a method of delivering medication to specific cells or tissues in the body, typically using specialized drug delivery systems or carriers that can selectively deliver drugs to the intended site of action. This approach can help minimize side effects and improve the efficacy of the medication, and is the focus of this study.

Drug delivery systems can be formulated using various materials, including polymers, lipids, and metals. The choice of material, such as hydrogel carrier, and its synthesis depends on the drug being delivered, the targeted site of action, and the desired mode of action [4]. Hydrogels have been used in drug delivery applications for several decades. The first reported use of hydrogels for drug delivery dates to the 1970s when hydrogel contact lenses were introduced for the extended war. Since then, hydrogels have been widely explored for various biomedical applications, including drug delivery. Over the years, significant advances have been developed for drug delivery with hydrogels, including the design of stimuli-responsive hydrogels that can release drugs in response to specific stimuli, such as changes in pH, temperature, or light [5]. Today, hydrogels are considered one of the most promising drug delivery platforms due to their biocompatibility, tunable properties, high water content, and versatility. The use of hydrogels has been successfully reported as a means of drug delivery for the treatment of inflammatory and infectious diseases in the fields of dentistry, ophthalmology, oncology, dermatology, rheumatology, and neurology, as well as in the treatment of intestinal diseases and wound healing [6,7,8,9].

Hydrogels are a class of materials that can absorb and retain large amounts of water, and they are commonly used for drug delivery applications due to their unique properties. Some of the different types of hydrogels used for drug delivery include natural hydrogel, synthetic hydrogel, self-healing hydrogels, and stimuli-responsive hydrogels. Natural hydrogels are made from natural polymers such as collagen, gelatin, chitosan, hyaluronic acid, and alginate. Synthetic hydrogels are synthesized from synthetic polymers, such as polyethylene glycol (PEG), polyvinyl alcohol (PVA), and poly(N-isopropylacrylamide) (PNIPAAm). Self-healing hydrogels can repair themselves after being damaged, which can help to extend their lifetime as drug delivery systems. Stimuli-responsive hydrogels can respond to a variety of stimuli, including pH, temperature, light, electric fields, and magnetic fields, and can be used for targeted drug delivery. pH-responsive hydrogels change their properties in response to changes in pH, which can be used to trigger drug release in response to changes in the local environment. Temperature-responsive hydrogels change their properties in response to changes in temperature, which can also be used to trigger drug release. In this study, a stimuli-responsive hydrogel is used and modified with different crosslinking agents. Some common hydrogels used in targeted drug delivery include polyethylene glycol (PEG), poly(N-isopropylacrylamide) (PNIPAAm), alginate, hyaluronic acid (HA), Poly(lactic-co-glycolic acid) (PLGA), and chitosan hydrogels. PEG is a widely used hydrogel material due to its biocompatibility and tunable properties. PEG hydrogels can be used for sustained drug release and can be engineered to be responsive to various stimuli. PNIPAAm is a thermo-responsive hydrogel that undergoes a reversible phase transition in response to changes in temperature. PNIPAAm hydrogels have been used for drug delivery applications that require triggered drug release in response to changes in temperature. Alginate is a natural polymer that can form hydrogels through ionic crosslinking. Alginate hydrogels have been used for drug delivery applications due to their biocompatibility and ability to protect drugs from degradation. HA is a natural polymer that is biocompatible and biodegradable. HA hydrogels have been used for targeted drug delivery due to their ability to target specific cell types through interactions with cell surface receptors. PLGA is a biodegradable polymer that has been widely used for drug delivery applications due to its ability to degrade into non-toxic byproducts and its tunable properties. PLGA hydrogels can be engineered for sustained drug release and can be used for targeted drug delivery. Chitosan is a natural polymer that is biocompatible and biodegradable. Chitosan hydrogels have been used for drug delivery applications due to their ability to release drugs in a sustained and controlled manner [10]. Chitosan is made from chitin—a natural polymer that is found in the shells of crustaceans, such as crabs, shrimps, and lobsters. Chitosan hydrogels have been widely studied for their potential applications in wound healing, tissue engineering, and drug delivery. Chitosan hydrogels have several properties that make them attractive for these applications. For example, they are biocompatible, biodegradable, and non-toxic, which makes them suitable for use in biological systems. They are also mucoadhesive, which means they can adhere to mucosal surfaces in the body, such as the lining of the gastrointestinal tract or the skin. Chitosan hydrogels have been used to promote wound healing in both animal models and human clinical trials. They have been shown to promote cell proliferation and migration to enhance the formation of new blood vessels, which is important for tissue regeneration. They enhance the absorption of drugs across mucosal surfaces, such as the gastrointestinal tract, due to their mucoadhesive properties. Chitosan hydrogels have been used for the delivery of a wide range of drugs, including small molecules, proteins, and nucleic acids. They can be prepared using a variety of techniques, including physical crosslinking, chemical crosslinking, and ionic gelation. In ionic gelation, chitosan is crosslinked with a polyanion such as sodium tripolyphosphate to form a hydrogel [5]. Further research is needed to optimize the properties of chitosan hydrogels and to explore their full potential in these applications. Therefore, this research focuses on preparing different types of chitosan hydrogels, i.e., linear hydrogel and hydrogels crosslinked with two crosslinkers, genipin and disulfide. Genipin is a small-molecule crosslinking agent that acts as a nontoxic crosslinker and can undergo self-polymerization under neutral conditions. Disulfide is a polymer–polymer crosslinking agent that has acute toxicity and is popular for in situ gel formation, where the reaction takes place under neutral conditions and provides mucoadhesion. Crosslinking in hydrogels is typically achieved through the formation of covalent or non-covalent bonds between polymer chains. The formation of covalent bonds between polymer chains in hydrogels can be investigated using density functional theory (DFT) calculations. Free radicals play an important role in the crosslinking process, which is generated by the initiator molecules used to initiate polymerization [11]. The reaction between free radicals and polymer chains affects the stability and properties of the resulting crosslinked network. However, the formation of covalent bonds between polymer chains is energetically favorable, and thus the resulting crosslinked network becomes stable and mechanically robust [11]. The non-covalent bonding interactions between polymer chains in hydrogels through hydrogen bonding are affected by different factors, such as temperature and pH, and play an important role in the formation, stability, and strength of hydrogels, among other properties [12]. Hydrogen bonding interactions can be tuned by adjusting the environmental conditions. Therefore, different crosslinking agents offer a design space to optimize various properties of hydrogels. Drug loading and release in chitosan hydrogels are important aspects of drug delivery that can be controlled by various factors, such as the type of drug, chitosan concentration, pH, temperature, and the presence of other additives [13,14]. The model drug ibuprofen has been investigated to observe the effects of chitosan concentration on drug loading and release, as well as the mechanism of drug release [15]. The drug loading efficiency increased with increasing chitosan concentration and the release of the drug was sustained over 24 h. It is reported that the release of the drug followed a diffusion-controlled mechanism, which was confirmed by fitting the release data into mathematical models. A similar investigation on chitosan hydrogel with the model drug doxorubicin analyzed the effect of pH on drug release [16]. The release of the drug was faster at lower pH values, followed by a combination of diffusion and erosion-controlled mechanisms. Therefore, drug-release kinetics can be controlled by adjusting the chitosan concentration and the pH of the release medium. Overall, these studies demonstrate the potential of chitosan hydrogel as a drug delivery system and provide insights into the factors that control drug loading and release. The findings on the drug loading and release efficacies of chitosan hydrogels modified with different crosslinkers for different drugs presented in this report could be useful in the design and optimization of chitosan-based drug delivery systems for various applications.

## 2. Results and Discussion

**Figure 1:**
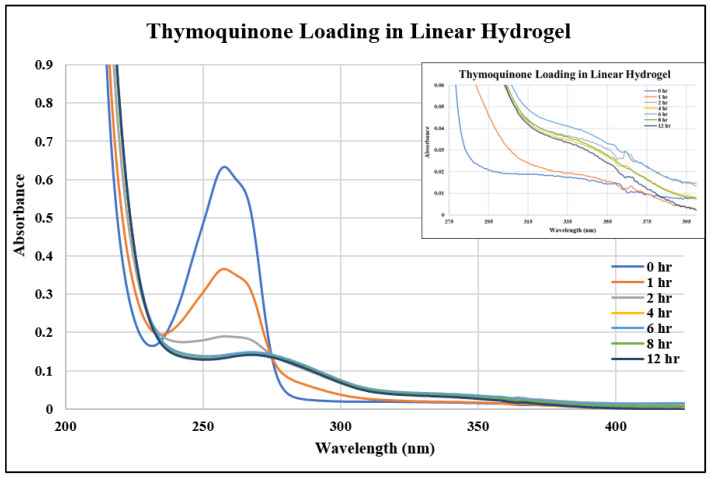
UV–Vis spectra of thymoquinone loading in linear hydrogel.

**Figure 2:**
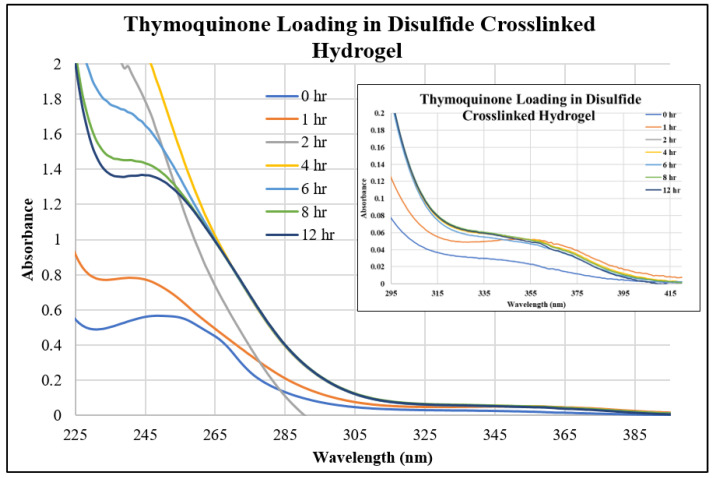
UV–Vis spectra of thymoquinone loading in disulfide-crosslinked hydrogel.

**Figure 3:**
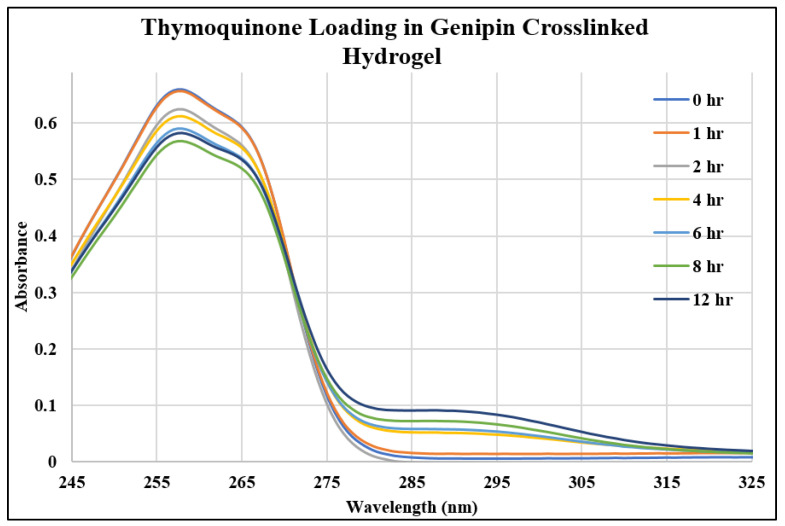
UV–Vis spectra of thymoquinone loading in genipin-crosslinked hydrogel.

**Figure 4:**
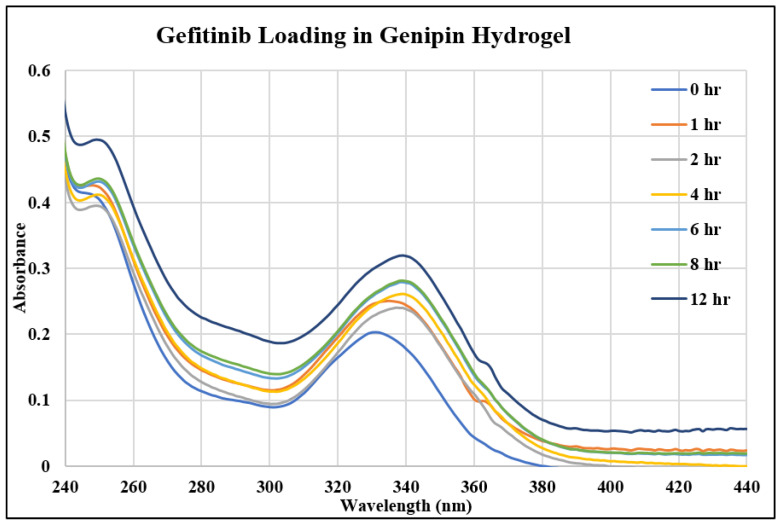
UV–Vis spectra of gefitinib loading in genipin-crosslinked hydrogel.

**Figure 5:**
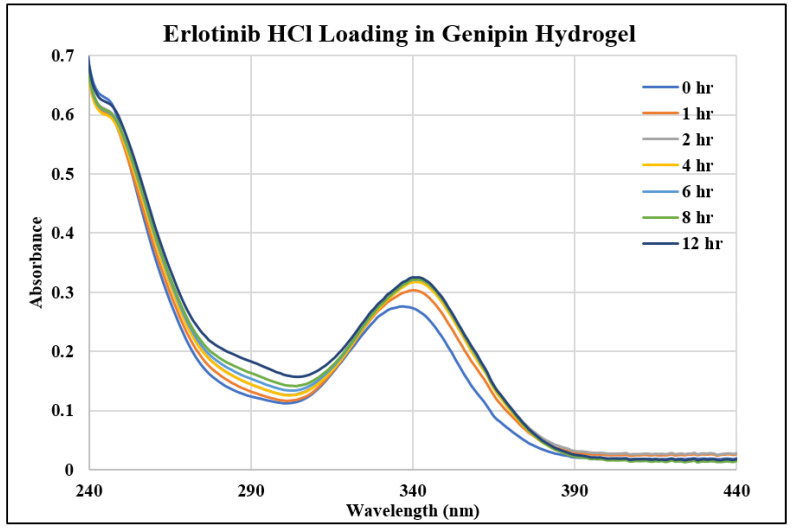
UV–Vis spectra of erlotinib HCl loading in genipin-crosslinked hydrogel.

In this study, we investigated three different drugs in three different hydrogels, resulting in the examination of nine unique systems for both loading and releasing, making eighteen different points of analysis. Figure 1, Figure 2 and Figure 3 show the UV–Vis spectra of the thymoquinone loading in different systems, i.e., the linear hydrogel, disulfide-crosslinked hydrogel, and genipin-crosslinked hydrogel, respectively. Supplementary Figure S1 depicts the absorption of the pure thymoquinone solution, and the peak occurs at 258 nm, which agrees well with Nadeem et al. [17]. The absorption at this peak for a pure solution is 0.74, whereas Figure 1 shows the peak occurring at the same wavelength just after submersing the gel into the drug solution at zero hours at a reduced absorption of 0.63. It is usual to have several peaks for a molecule depending on different bonds, and the peaks can be different for different solvents; however, the peak shifts to different wavelengths for different concentrations for this drug molecule, as shown in Figure 1, for longer times. When the linear hydrogel absorbs the drug molecules from the drug solution of de-ionized (DI) water at different times, the drug concentration is lowered in the solution and the absorbance is lowered. These lowered peaks are observed to shift to the right at 268 nm wavelength. This shifting behavior for this thymoquinone drug with different systems was observed by Nadeem et al. as well; however, their shifted wavelengths and peak absorptions were different because the interacting materials were different. A second peak is observed at 355 nm, which is not prominent for this system; however, Figure 2, depicting thymoquinone loading in disulfide-crosslinked gel, shows this peak, as highlighted in the inset. Figure 2 depicts the different behavior of thymoquinone drugs with disulfide-crosslinked gel. Thymoquinone was loaded in the linear gel gradually demonstrated by a higher initial peak of the remnant. The peaks decreased over time, meaning the drugs were absorbed in the gel system, thus leaving less concentration in the remnant. The initial peak is found at a lower value for thymoquinone loading in the disulfide-crosslinked system at zero hours, occurring at 248 nm wavelength, and the peak is found to increase at 1 h to 240 nm wavelength. This indicates the drug was loaded in the gel rapidly at zero hours and returned to the solution when submersed for a longer time; therefore, the peak at 1 h is higher than the initial peak. Interestingly, due to the interactions of the drug, hydrogel, and solvent, higher values of absorbance are observed at this wavelength for longer times, but these peaks are ignored for the analysis in this study. However, the second peak, found at 355 nm for this drug, shifted to the right with a higher wavelength and lower absorption, as shown in the inset of Figure 2. This can be used for the release analysis of lower concentrations. However, to be consistent in the comparison with different drugs and gels, the first two peaks found at 248 nm are used for the analysis of loading in this disulfide-crosslinked system. The initial loading of thymoquinone in the genipin-crosslinked system shows a higher initial peak of the remnant at 258 nm wavelength, which decreases gradually over time, meaning the drug loading is steady and the concentration accumulates over time for this system, as shown in Figure 3. However, the absorption trend shifted the opposite way after 271 nm wavelength. Figure 3 shows that the absorption spectra for the same concentrations is lower at zero hours after 271 nm and gradually increases over time, which is the opposite to that shown at 258 nm. Different bonds absorb light of different wavelengths, and their amplitude of atomic vibration during energy absorption at different energy states may exhibit underdamped, overdamped, or critically damped systems. Therefore, the trend of amplitude increment and decrement could be different at different wavelengths for the same concentration. The Beer-Lambert law calculates the concentration of a molecule, assuming the light absorption is proportional to the molar concentration and path length the light travels, and that the proportionality is constant. However, the constant known as the molar absorption coefficient is different at different wavelengths if different peaks are found for different wavelengths. Moreover, if a molecule shows peak shifting behavior, the concentration profile does not adopt the Beer–Lambert law, as reported by [18]. Tong et al. formulated numerous solutions with varying concentrations and observed the peak profiles against logarithmic concentrations for shifting peaks of absorption, which is beyond the scope of this study. Therefore, we focused on analyzing the drug loading and release characteristics of these developed gels by the ratio of absorption (A) to the initial drug concentration $\left(C_{o}\right)$, i.e., $\frac{A}{C_{0}}$. A higher value for this parameter means higher drug molecules in the solution. Therefore, for the loading analysis, a lower value of this parameter means more drugs have been loaded to the gels, and for the release analysis, a higher value of this parameter means more drugs have been released to the solutions. The loadings of gefitinib and erlotinib HCl in genipin-crosslinked systems are shown in Figure 4 and Figure 5, respectively. Two peaks are found according to these two figures: one at 249 nm and the other one at 330 nm. Both of these drugs have a shifting behavior as well. The rest of the loading and release spectra are shown in Supplementary Figures S2–S14. The loading was conducted with DI water, and the release study was conducted with a phosphate-buffered solution. The release spectra exhibit very low absorption; however, in the human body context, the gel would break down itself to deliver the drugs [19].

The molecular weights of thymoquinone, gefitinib, and erlotinib hydrochloride are 164.2 g/mol, 446.9 g/mol, and 429.9 g/mol, respectively. The no. of moles was calculated by dividing the total weight of the drug (10 mg) by the corresponding molecular weight. Thus, the concentrations of thymoquinone, gefitinib, and erlotinib were calculated as 60.9 µM, 22.4 µM, and 23.2 µM, respectively. To analyze and compare the performances of different drugs in different systems, the $\frac{A}{C_{0}}$ parameters for different drugs in differently prepared chitosan hydrogels were plotted. The maximum absorbance data for the thymoquinone drug in the linear hydrogel at different times, i.e., 0 h, 1 h, 2 h, 4 h, 6 h, 8 h, and 12 h, were taken, and $\frac{A}{C_{0}}$ parameters were calculated and compared with similar data for erlotinib and gefitinib in all three hydrogels. As different peaks were found at different wavelengths for different systems, the peaks that occurred at closer wavelengths were taken for comparison, as the absorption coefficients were different at different wavelengths. Peaks were collected at 258 nm for thymoquinone, 249 for gefitinib, and 248 for erlotinib for loading. The release absorptions were very low, and peaks for release were taken at 230 nm for thymoquinone, 249 for gefitinib, and 247 for erlotinib. The peaks for all drug molecules found in this study agree well with other studies [20,21,22].

**Figure 6:**
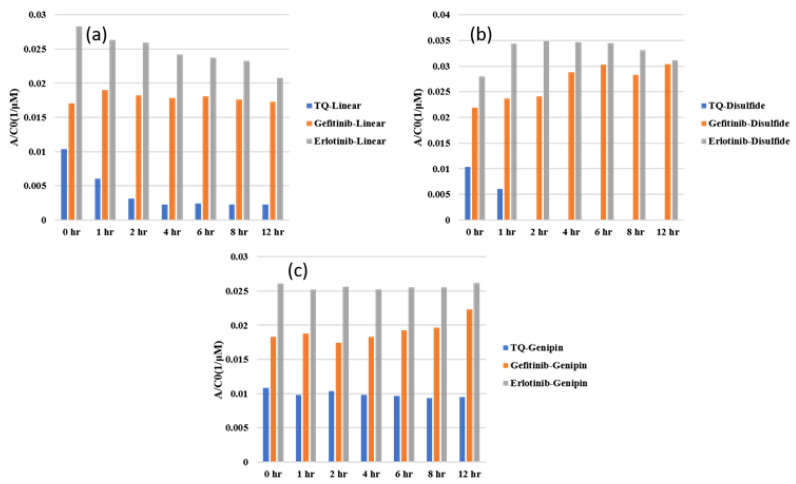
Comparison of loading of different drugs in different systems: (**a**) linear hydrogel, (**b**) disulfide-crosslinked hydrogel, and (**c**) genipin-crosslinked hydrogel.

Figure 6a indicates that the $\frac{A}{C_{0}}$ parameter of thymoquinone in the linear hydrogel decreases over time for 2 h, and then becomes constant. Thus, the loading rate of thymoquinone in linear hydrogel is steady, and it takes two hours to reach maximum loading. A similar trend is found for erlotinib in the linear gel; however, it keeps loading for 12 h, meaning it would take a longer time to load to the maximum capacity. Thymoquinone is a small molecule with a lower molecular weight, as well as a lower polar surface area (PSA). Low molecular weight and low PSA provide higher permeability. The PSA values for thymoquinone, gefitinib, and erlotinib are $34.1\;A^{2}$, $68.7\;A^{2}$, and $74.7\;A^{2}$. However, the amount of drug loaded is higher for erlotinib than thymoquinone due to its higher PSA that offers more interaction sites to the carrier gel to adhere with. On the other hand, gefitinib’s loading capacity is in between thymoquinone and erlotinib, as its PSA is in between them. Figure 6b shows a similar trend for thymoquinone in a disulfide-crosslinked system; however, gefitinib and erlotinib show opposite trends. The initial loading of the drug is higher at the first hour, meaning the drugs are loaded to their maximum capacities rapidly and then return to the solution as they did not find it favorable. The initial high loading could be due to the 3D porous structure of the disulfide-crosslinked system; however, the rigidly bonded sulfur molecule was not found favorable for the covalently bonded small drug molecules of gefitinib and erlotinib. However, this effect could not alter thymoquinone’s behavior, as its mobility is relatively high compared to the two other drugs due to its very low molecular weight and PSA. Figure 6c shows erlotinib and thymoquinone showing almost steady and constant loading throughout the whole period, meaning they are loaded to the maximum capacity at the beginning and are stable for a longer time. This is because genipin is a covalently bonded small biomolecule that offers favorable sites for covalently bonded drug molecules. Even though gefitinib’s loading capacity in the disulfide-crosslinked system is in between thymoquinone and erlotinib due to its molecular weight and PSA, it did not find the conditions very favorable, and diffused out for 6 h and then fluctuated. A detailed study of the molecular behavior of gefitinib may lead to a better understanding of this reason. Depending on the target site, the loading rate can be controlled effectively, for example to achieve an initial slower/higher rate, or stability over the whole period. However, all three figures show that erlotinib has the highest loading capacity of all three types of gel, whereas thymoquinone shows the lowest capacity and gefitinib lies between the two. In terms of loading rate, thymoquinone is loaded rapidly to its maximum capacity.

**Figure 7:**
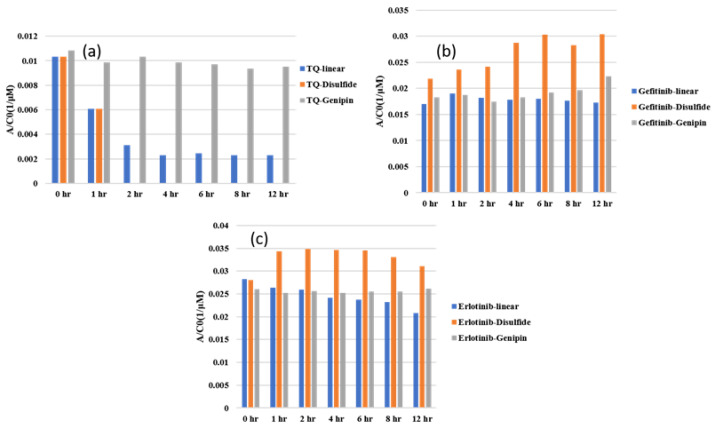
Comparison of loading of different drugs in different systems: (**a**) thymoquinone, (**b**) gefitinib, and (**c**) erlotinib HCL.

**Figure 8:**
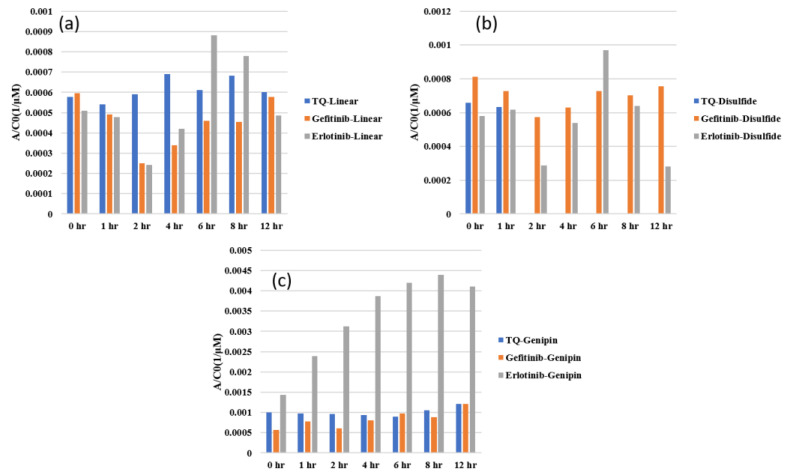
Comparison of release of different drugs in different systems: (**a**) linear hydrogel, (**b**) disulfide-crosslinked hydrogel, and (**c**) genipin-crosslinked hydrogel.

**Figure 9:**
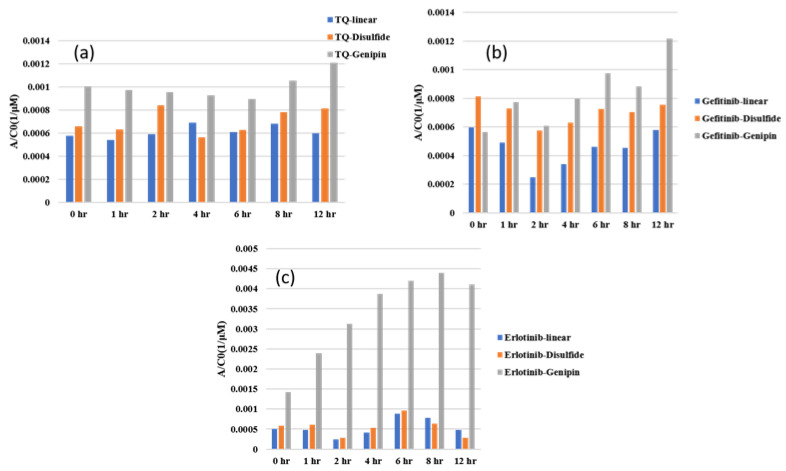
Comparison of release of different drugs in different systems: (**a**) thymoquinone, (**b**) gefitinib, and (**c**) erlotinib HCL.

Figure 7a shows the loading data of thymoquinone in three different types of gel, and indicates that thymoquinone loading in linear chitosan happens over the whole period, and is stable for genipin for the whole time. Gefitinib and erlotinib are both less favorable in disulfide-crosslinked systems, as depicted in Figure 7b,c. In addition, disulfide shows toxicity, which is not a problem for genipin crosslinking agents. Therefore, disulfide is not recommended based on this study. Figure 8a–c show the UV–Vis data during release. In this case, the higher the $\frac{A}{C_{0}}$ value, the higher the releasing capacity. Thymoquinone is released to the maximum capacity for 4 h, and then fluctuates. Erlotinib fluctuates initially; however, it is released to the maximum capacity in 6 h, and its releasing capacity is higher than the two others. Figure 8c shows that the release of all drugs in disulfide is higher initially. As was mentioned earlier, disulfide does not offer a good site for the drugs. Erlotinib shows stable release to the highest capacity over the given period, showing a higher rate than the two other drugs, as depicted in Figure 8c. Figure 9a–c depict the drug release data of three drugs in three systems, exhibiting that genipin-crosslinked systems show a higher release rate regardless of the drug. The physical interactions among drug molecules and gels are not observed closely in this study. The higher mobility of thymoquinone and the better compatibility of the genipin crosslinking agent agree well with our previously published study using molecular dynamics simulations [23]. This experimental study demonstrates that the customization of chitosan structures with crosslinking agents improves therapeutic results, and can minimize side effects by regulating drug-release characteristics sensitive to bodily situations [24]. Molecular weight, diffusivity, the morphology of the carrier, and surface charge play important roles in the sustained rate of loading and releasing, as we have discussed in this report. In addition to the study of biocompatibility and formulations, several other factors, such as cytotoxicity, cell viability, the potential application of hydrogel to living tissues, efficacy, safety for long-term stability, and degradation behavior, as well as clinical trials, need to be investigated before administration to the human body [25,26,27,28,29].

## 3. Conclusions

Thymoquinone consistently exhibited higher diffusivity, meaning it was loaded rapidly; however, its loading capacity was the lowest among the three drugs. This behavior applies to all hydrogel systems studied in this report. The loading and release capacities of gefitinib were in between thymoquinone and erlotinib, on average. Erlotinib shows higher loading and releasing capabilities; however, its release fluctuates in linear and disulfide-crosslinked systems (it showed stable release in the genipin-crosslinked system). Genipin-crosslinked systems were found to be favorable for all drugs. On the other hand, disulfide-crosslinked systems were not favorable for any of the drugs as studied. The chitosan hydrogels modified with different crosslinkers in this study have huge prospects in tissue engineering [30]. However, investigations into swelling ratios [31], as well as the degree of crosslinking, their cytotoxicity, and biodegradation, are left for future studies. The linear gels were found as individual separated grains, whereas elastic solids were obtained after modifying the chitosan with the crosslinking agents that confirmed the crosslinking. However, a detailed study of the developed gels using Fourier Transform Infrared Spectroscopy (FTIR), scanning electron microscopy (SEM), stability methods, and rheology was not conducted in this report due to limited resources, and is left for future studies.

## 4. Materials and Methods

**Figure 10:**
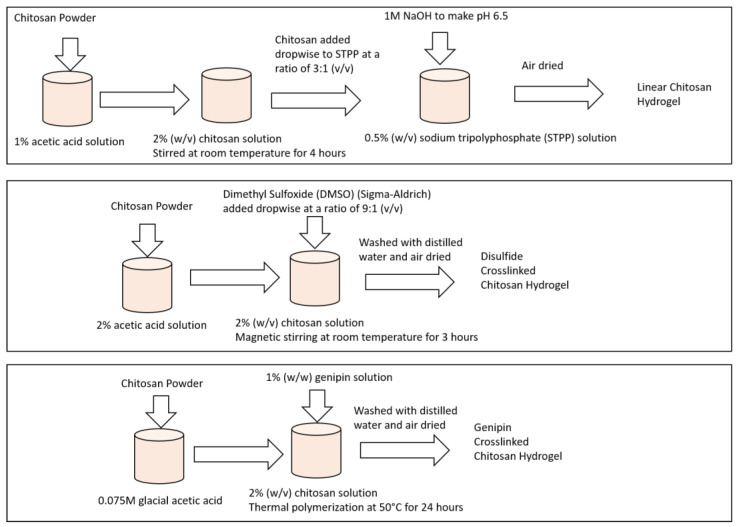
Sample preparation methodology.

Figure 10 depicts the sample preparation method. A chitosan-based hydrogel with various crosslinkers is typically prepared in a multi-step procedure for targeted drug delivery. The proper quantity of chitosan powder was weighed and added to a container for chitosan dissolution. The chitosan was stirred in a suitable solvent, such as glacial acetic acid, until completely dissolved. Sodium hydroxide (NaOH) was used to adapt the pH of the chitosan solution to the required range. The appropriate crosslinking agent was added to the chitosan solution depending on the crosslinking mechanism desired, i.e., covalent or ionic. A sodium tripolyphosphate (STPP) suspension was used for ionic crosslinking. The crosslinker was then added to the chitosan solution dropwise while swirling, until gelation occurred. Genipin was used for ionic crosslinking. The crosslinking solution was added dropwise while stirring to the chitosan solution, which was prepared individually. The crosslinked chitosan solution was given time to rest for gelation and hydrogel formation. Cross-linked chitosan can absorb water without dissolution. The gelation time depends on the type and amount of the crosslinker utilized. The chitosan hydrogel was submerged in the medication solution to facilitate drug loading into the hydrogel network. The amount of drug loaded in the hydrogel was dictated by the loading circumstances, hydrogel type, and time. After adding drugs, the hydrogel was washed with deionized water or a suitable buffer solution to eliminate any excess or unbound drug molecules. The hydrogels loaded with drugs were then immersed in the solution again to release the drugs to analyze releasing efficacy. Drug loading and release tests were performed in physiologically realistic environments, such as phosphate-buffered saline (PBS), to assess their controlled loading and release capabilities.

### 4.1. Sample Preparation

#### 4.1.1. Preparation of Linear Chitosan Hydrogel Sample

Initially, chitosan powder with a molecular weight of 190,000 Da (Sigma-Aldrich, Milwaukee, WI, USA) was dissolved in a 1% acetic acid solution, resulting in a 2% (*w*/*v*) chitosan solution to prepare the linear chitosan hydrogel. This solution was stirred at room temperature for 4 h until a homogeneous mixture was achieved. Simultaneously, a sodium tripolyphosphate (STPP) solution was prepared by dissolving STPP (Sigma-Aldrich, Milwaukee, WI, USA) in distilled water, yielding a concentration of 0.5% (*w*/*v*). The pH of the STPP solution was adjusted to 6.5 using 1 M NaOH. Next, the chitosan solution was gradually added dropwise to the STPP solution under continuously stirring at room temperature. The chitosan to STPP ratio was maintained at 3:1 (*v*/*v*). The resulting mixture was allowed to react for 30 min at room temperature, leading to the formation of a complex and the subsequent hydrogel formation through the interaction between the chitosan and the STPP. Following the reaction, the hydrogel was washed with distilled water to eliminate any unreacted STPP and acetic acid. Finally, the hydrogel was air-dried to obtain the chitosan hydrogel in solid form [32].

#### 4.1.2. Preparation of Disulfide-Crosslinked Chitosan Hydrogel Sample

The preparation process involved several steps. Firstly, the chitosan solution was prepared by dissolving chitosan powder (MW 190,000 Da, Sigma-Aldrich) in a 2% acetic acid solution with a concentration of 2% (*w*/*v*). The mixture was stirred using a magnetic stirrer for 3 h until it became clear. Simultaneously, a disulfide crosslinker solution, dimethyl sulfoxide (DMSO) (Sigma-Aldrich, Milwaukee, WI, USA), was added dropwise at a ratio of 9:1 (*v*/*v*) and stirred for 30 min at room temperature to facilitate crosslinking. The resulting mixture was then poured into a mold and left to gel for 2 h at room temperature. Afterward, the chitosan hydrogel was washed with distilled water to remove any unreacted crosslinker. Following this, the chitosan hydrogel was air-dried in a vacuum chamber for 24 h. Finally, the dried chitosan hydrogel was subjected to characterization to assess its physical and chemical properties.

#### 4.1.3. Preparation of Genipin-Crosslinked Chitosan Hydrogel Sample

Genipin-crosslinked chitosan hydrogels were synthesized via thermal free-radical polymerization. To initiate the process, chitosan was dissolved in 0.075 M glacial acetic acid, reaching a final concentration of 2% (*w*/*v*). Subsequently, a 1% (*w*/*w*) genipin (Sigma-Aldrich, Milwaukee, WI, USA) solution was used as a chemical crosslinker for thermal polymerization, which occurred at 50 °C for 24 h. The chitosan hydrogel beads were then washed with distilled water to remove any unreacted genipin. The chitosan hydrogel beads were air-dried for over 24 h. The dried chitosan hydrogel beads were then characterized for their physical and chemical properties [13,14].

**Figure 11:**
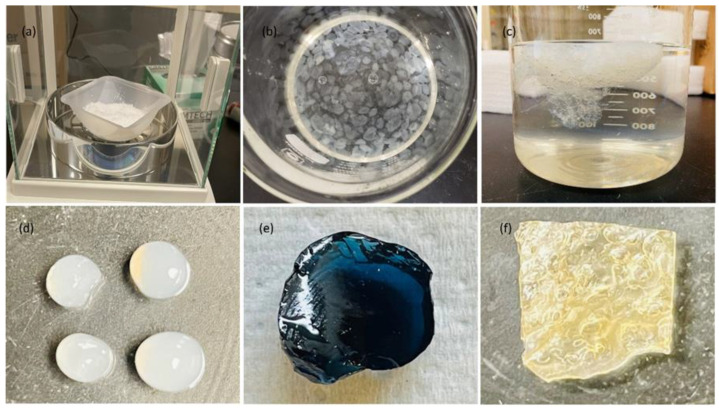
(**a**) Chitosan powder; (**b**) formation of beads in linear chitosan; (**c**) spongy gel with disulfide crosslinker; (**d**) final sample of linear chitosan; (**e**) final sample of genipin-crosslinked chitosan; and (**f**) final sample of disulfide-crosslinked chitosan.

Figure 11 shows the final samples prepared for the drug loading and release characterizations.

### 4.2. Drug Selection

As mentioned earlier, three drugs are studied in this report and their chemical structures are shown in the Supplementary Files in Figure S15.

#### 4.2.1. Thymoquinone

In recent years, there has been an increasing interest in naturally occurring phytochemical compounds (found in plants that protect plants against bacteria, viruses, and fungi) that possess potential anti-cancer properties. This interest stems from their relative non-toxicity, cost-effectiveness, and availability in ingestible forms. It is noteworthy that more than 25% of drugs utilized in the past two decades have been directly derived from plants, and an additional 25% are chemically modified natural products. Among these natural compounds, the black seed (Nigella sativa, from the Ranunculaceae family) has gained particular attention. This annual herb is known by various names, including the black caraway seed and “the Blessed Seed”, and it thrives in countries neighboring the Mediterranean Sea, as well as Bangladesh, India, and Pakistan [33,34]. Thymoquinone is a phytochemical compound found in the seeds of the Nigella sativa (black cumin) plant. It has been studied for its potential medicinal properties, including anti-inflammatory, antioxidant, and anticancer effects. Thymoquinone has been shown to have cytotoxic effects on a variety of cancer cell lines, including breast cancer, prostate cancer, and pancreatic cancer.

#### 4.2.2. Erlotinib Hydrochloride

Erlotinib hydrochloride is an important medication used in cancer treatment, specifically for certain types of lung cancer and pancreatic cancer. It is an epidermal growth factor receptor (EGFR) tyrosine kinase inhibitor (TKI) that inhibits the signaling pathways involved in cancer cell growth and proliferation. In non-small cell lung cancer (NSCLC), erlotinib hydrochloride has shown efficacy, particularly in patients with specific EGFR mutations. Studies have demonstrated improved progression-free survival and overall survival in NSCLC patients treated with erlotinib hydrochloride compared to conventional chemotherapy. It has become an important targeted therapy option, particularly for patients with EGFR mutation-positive NSCLC. Additionally, erlotinib hydrochloride has also shown clinical benefit in the treatment of pancreatic cancer. Clinical trials have demonstrated improved overall survival in patients receiving erlotinib hydrochloride in combination with gemcitabine, a standard chemotherapy drug for pancreatic cancer [35,36]. The use of erlotinib hydrochloride in cancer treatment is supported by various clinical studies and has been approved by regulatory authorities for specific indications. However, it is important to note that the efficacy and suitability of erlotinib hydrochloride may vary depending on individual patient characteristics and cancer type. Therefore, it is crucial to consult with a healthcare professional for personalized treatment recommendations.

#### 4.2.3. Gefitinib

Gefitinib is a small-molecule tyrosine kinase inhibitor that is used in cancer treatment, particularly for non-small cell lung cancer (NSCLC). It targets and inhibits the epidermal growth factor receptor (EGFR) tyrosine kinase, which is overexpressed in many cancer cells. Gefitinib has been studied for its potential use in targeted drug delivery using chitosan hydrogel as a carrier [37,38]. Li et al. (2016) prepared a chitosan hydrogel loaded with gefitinib for sustained release in cancer treatment [37]. The chitosan hydrogel was prepared using a method that involved the in situ crosslinking of chitosan and β-glycerophosphate. Gefitinib was then incorporated into the hydrogel by simple mixing. The hydrogel was then characterized for its drug-release properties and the results showed the sustained release of gefitinib over 24 h.

### 4.3. Ultraviolet–Visible (Uv–Vis) Spectroscopy

We used ultraviolet–visible (UV–Vis) technology to analyze the drug loading and release tendencies of chitosan hydrogels in this study. To study drug loading, the drugs and chitosan hydrogels were mixed and allowed to equilibrate. A 100 mL volumetric flask was used to prepare 0.01 g/L drug solution. The mixture was then centrifuged to separate the hydrogel from the solution. Then, 1 gm of chitosan was added to 100 mL of 2.5% acetic acid. A stirring bar was placed to mix the content. Hydrogels were collected using a filter. The supernatant can be analyzed using UV–Vis spectroscopy to determine the presence of drugs in the solution. To study drug release, the drug-loaded chitosan hydrogel can be immersed in a buffer solution and kept at a constant temperature. Drug loading was analyzed using de-ionized water; however, drug release was studied with clear phosphate-buffered solution (PBS) at room temperature in a 100 mL volumetric flask. The buffer solution was periodically sampled to a 1 cm cuvette and analyzed using UV–Vis spectroscopy at different time points. The volume change was neglected in this study. Charting the quantity of drugs given over time will reveal the drug’s release kinetics [39,40]. Therefore, the UV–Vis spectroscopy absorbance data of the nine different supernatant samples investigated in this study are used to compare drug loading and releasing efficacies.

## Data Availability

The data presented in this study are openly available in article.

## Supplementary Materials

The following supporting information can be downloaded at: https://www.mdpi.com/article/10.3390/gels10070421/s1, Figure S1: UV–Vis spectra of thymoquinone solution in DI water; Figure S2: UV–Vis spectra of gefitinib loading in linear hydrogel; Figure S3: UV–Vis spectra of gefitinib loading in disulfide crosslinked hydrogel; Figure S4: UV–Vis spectra of erlotinib HCL loading in linear hydrogel; Figure S5: UV–Vis spectra of erlotinib HCL loading in disulfide crosslinked hydrogel; Figure S6: UV–Vis spectra of thymoquinone releasing in linear hydrogel; Figure S7: UV–Vis spectra of thymoquinone releasing in disulfide crosslinked hydrogel; Figure S8: UV–Vis spectra of thymoquinone releasing in genipin crosslinked hydrogel; Figure S9: UV–Vis spectra of gefitinib releasing in linear hydrogel; Figure S10: UV–Vis spectra of gefitinib releasing in disulfide crosslinked hydrogel; Figure S11: UV–Vis spectra of gefitinib releasing in genipin crosslinked hydrogel; Figure S12: UV–Vis spectra of erlotinib HCl releasing in linear hydrogel; Figure S13: UV–Vis spectra of erlotinib HCl releasing in disulfide crosslinked hydrogel; Figure S14: UV–Vis spectra of erlotinib HCl releasing in genipin crosslinked hydrogel; Figure S15: Chemical Structures of three drug molecules used in this study [34,36,38]; Table S1: Absorbance while loading; Table S2: Absorbance while loading.
