# Supplementary material for: Effect of Crosslinking Agents on Chitosan Hydrogel Carriers for Drug Loading and Release for Targeted Drug Delivery
Source: Gels. 2024 Jun 26;10(7):421. doi: 10.3390/gels10070421 (PMC11276364; doi:10.3390/gels10070421)
Supplement: Supplementary file 1 [file gels-10-00421-s001.zip › gels-3049228-supplementary.pdf]

Supplementary Information

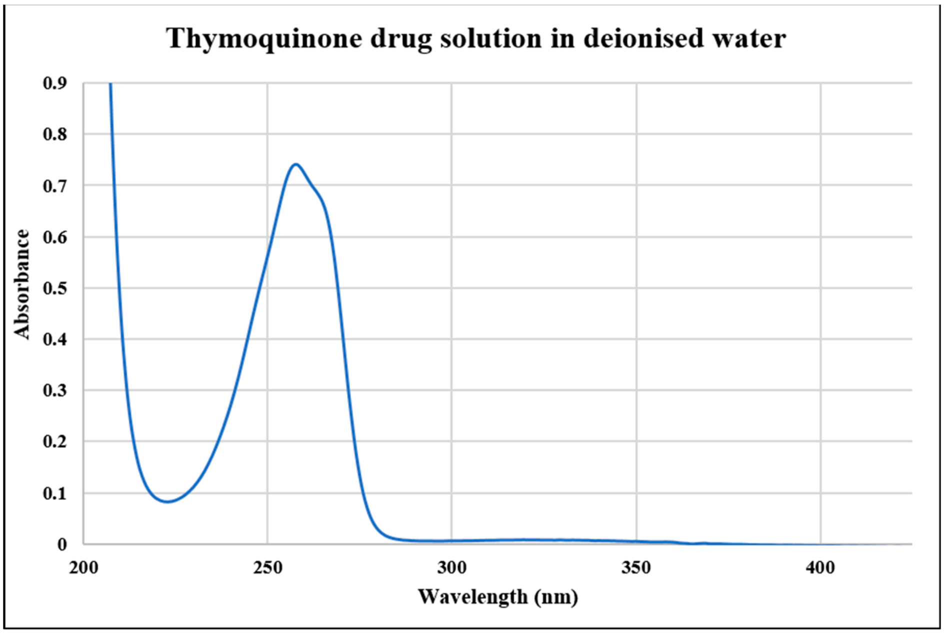

Figure S1: UV-Vis spectra of thymoquinone solution in DI water.

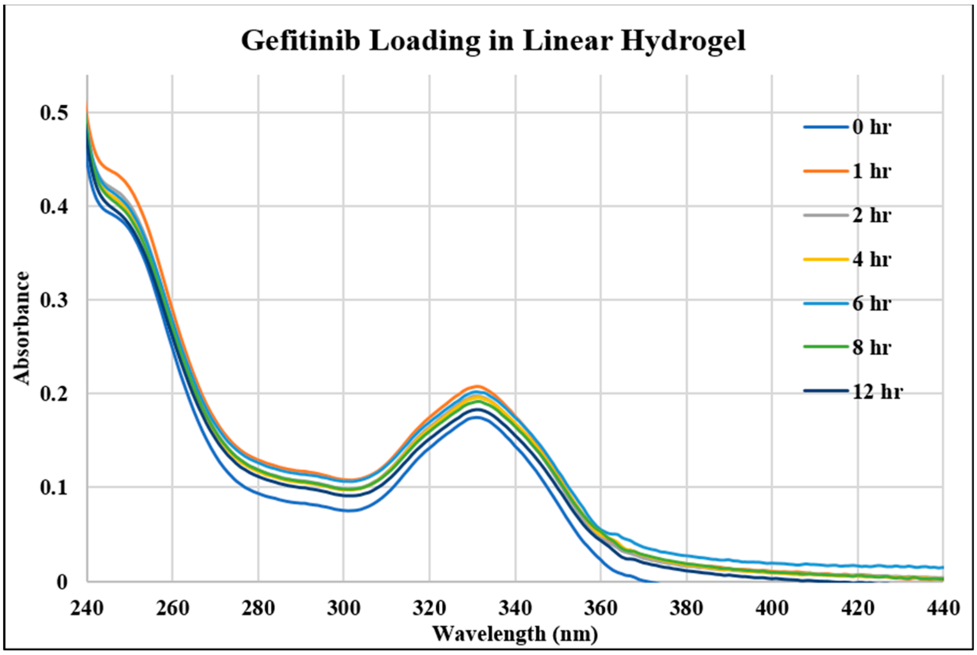

Figure S2: UV-Vis spectra of gefitinib loading in linear hydrogel.

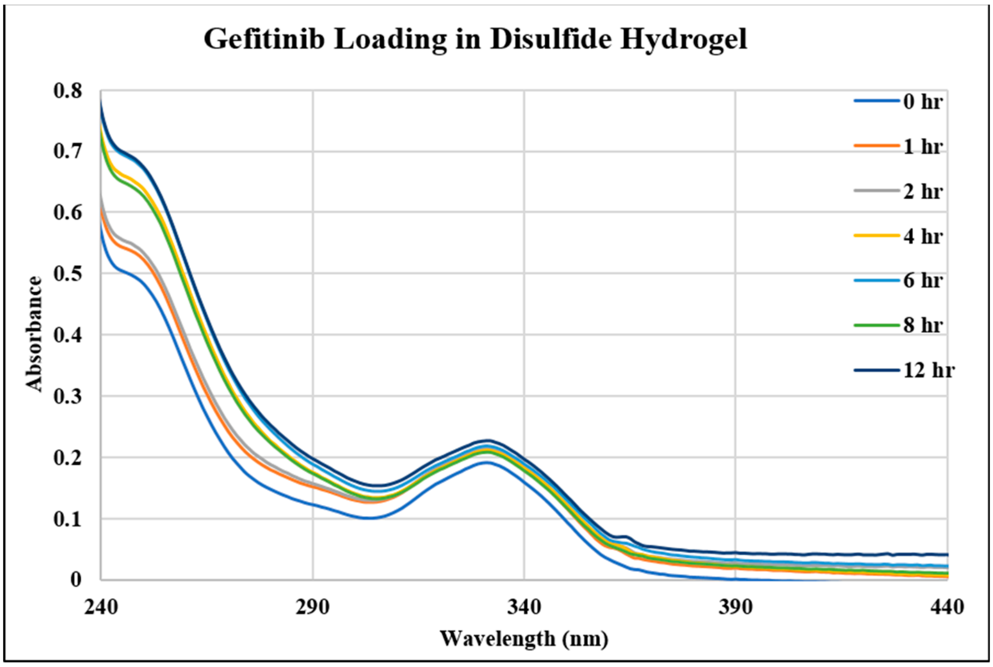

Figure S3: UV-Vis spectra of gefitinib loading in disulfide crosslinked hydrogel.

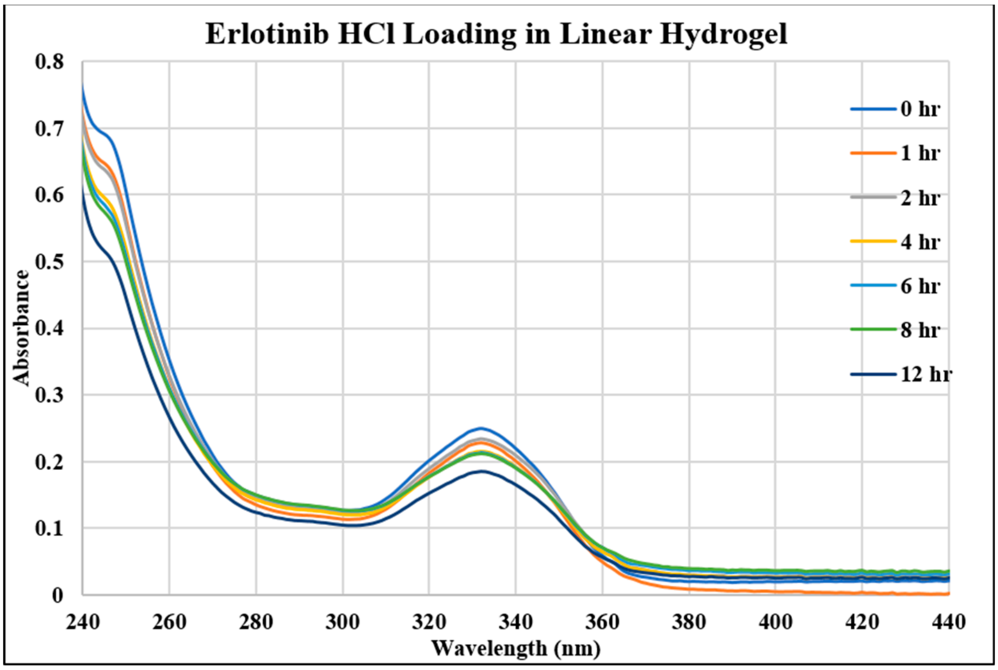

Figure S4: UV-Vis spectra of erlotinib HCL loading in linear hydrogel.

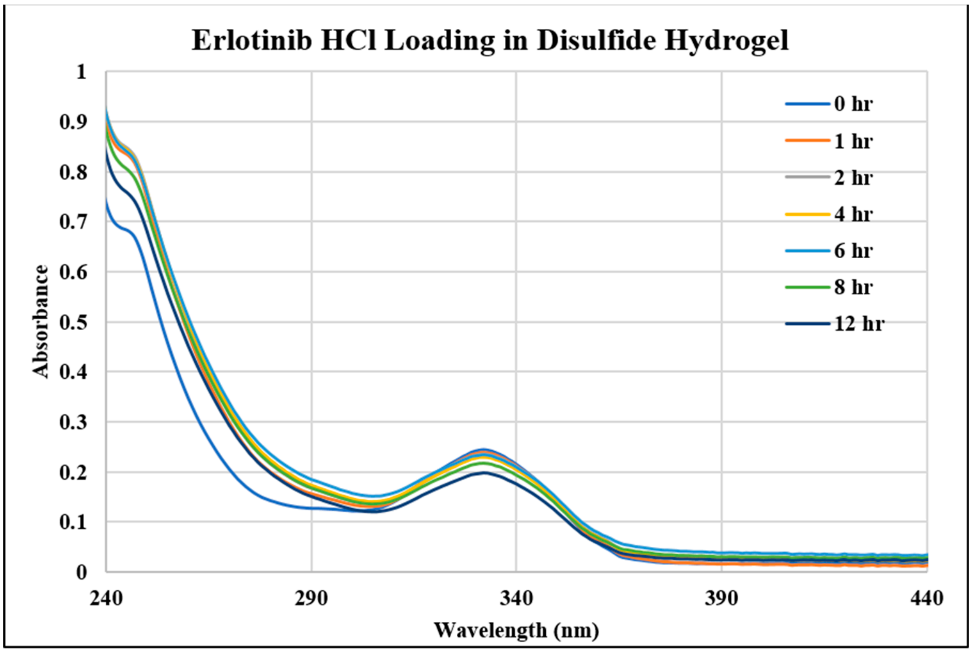

Figure S5: UV-Vis spectra of erlotinib HCL loading in disulfide crosslinked hydrogel.

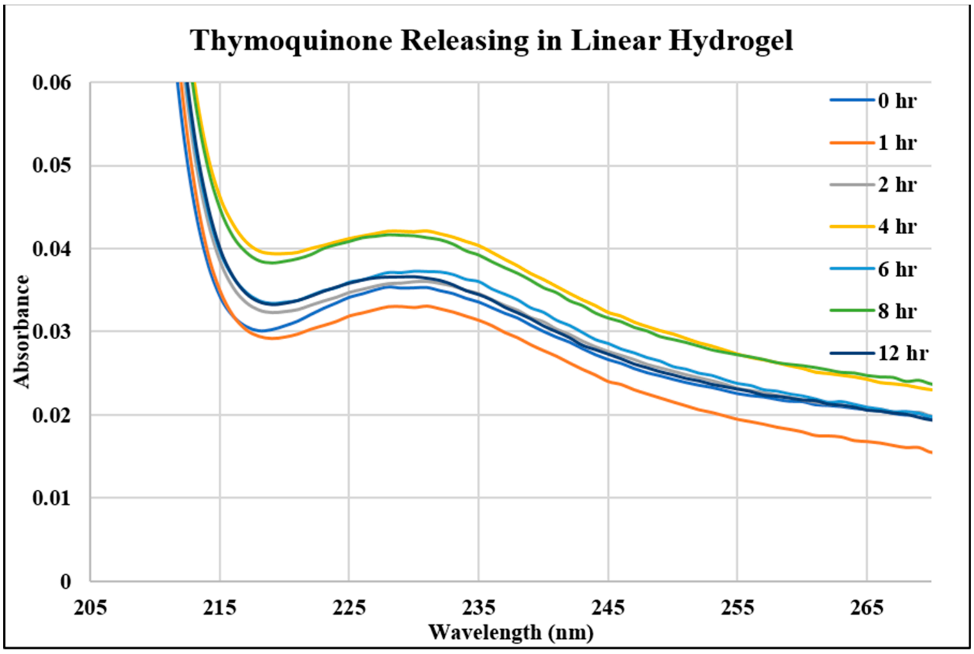

Figure S6: UV-Vis spectra of thymoquinone releasing in linear hydrogel.

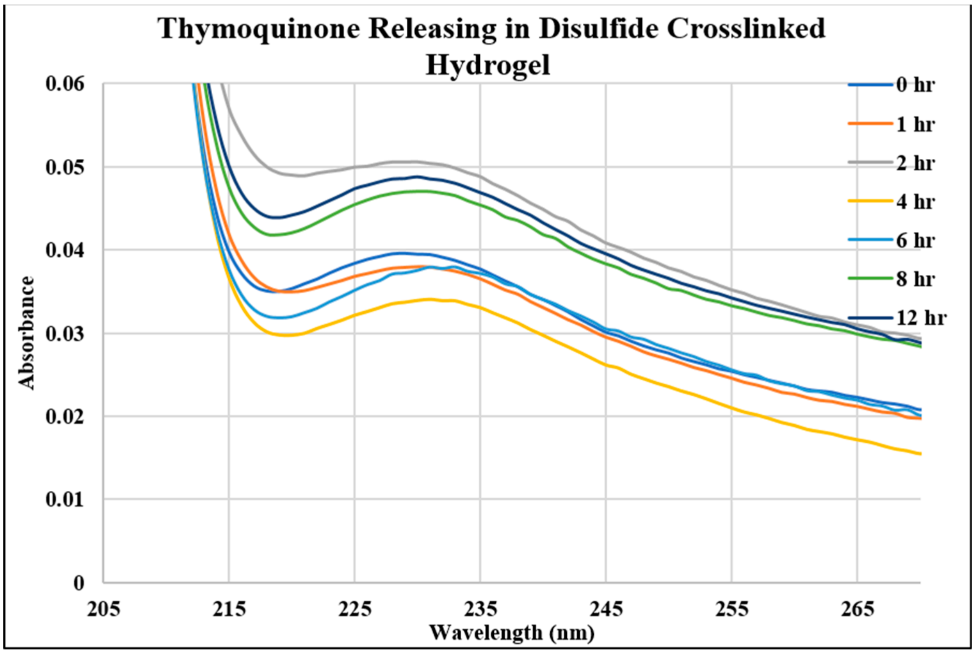

Figure S7: UV-Vis spectra of thymoquinone releasing in disulfide crosslinked hydrogel.

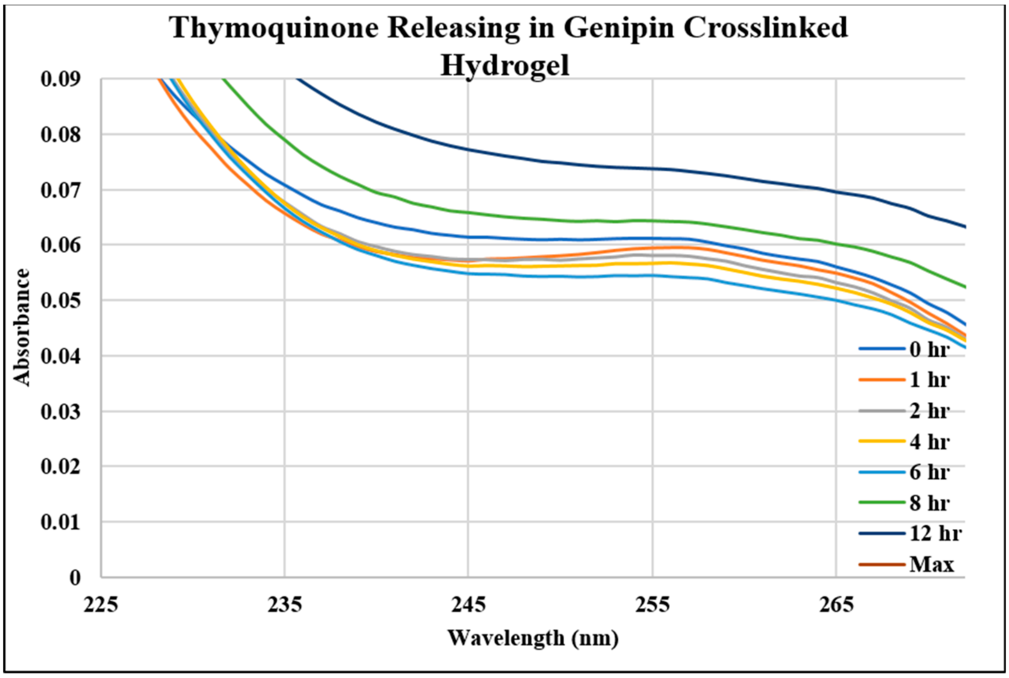

Figure S8: UV-Vis spectra of thymoquinone releasing in genipin crosslinked hydrogel.

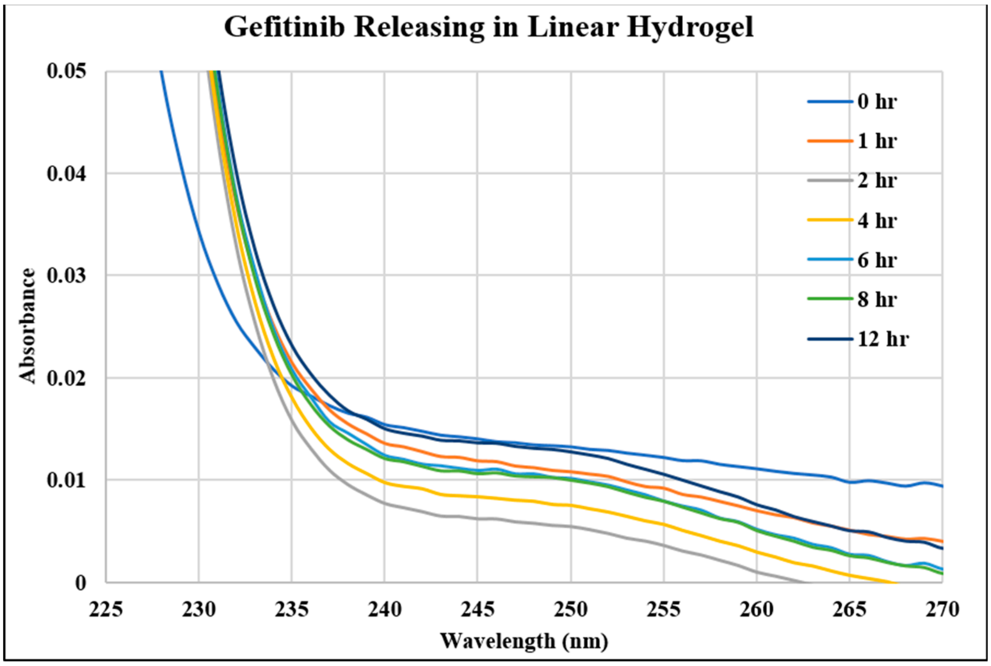

Figure S9: UV-Vis spectra of gefitinib releasing in linear hydrogel.

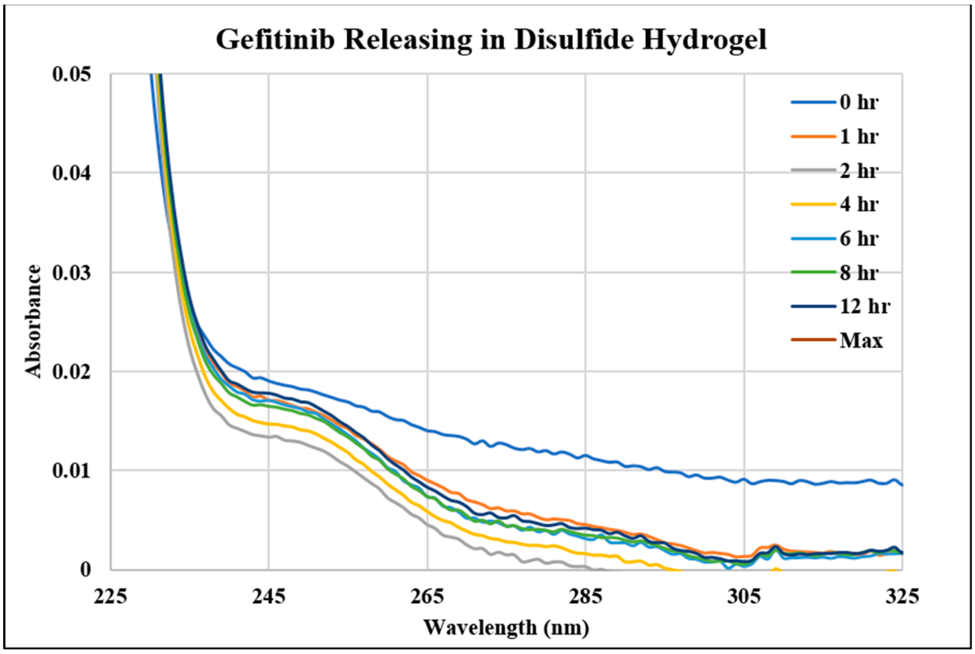

Figure S10: UV-Vis spectra of gefitinib releasing in disulfide crosslinked hydrogel.

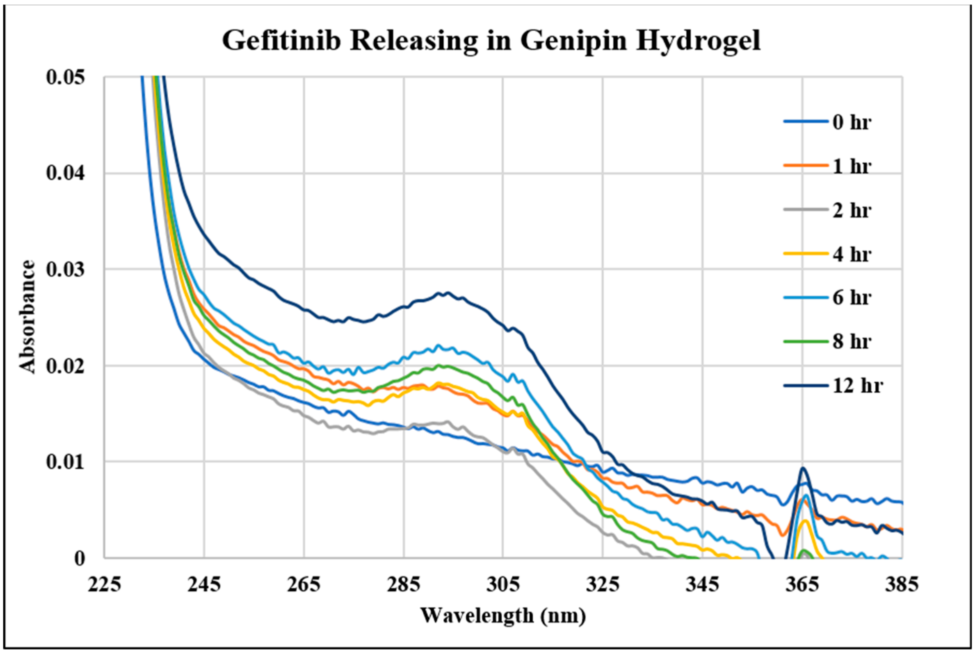

Figure S11: UV-Vis spectra of gefitinib releasing in genipin crosslinked hydrogel.

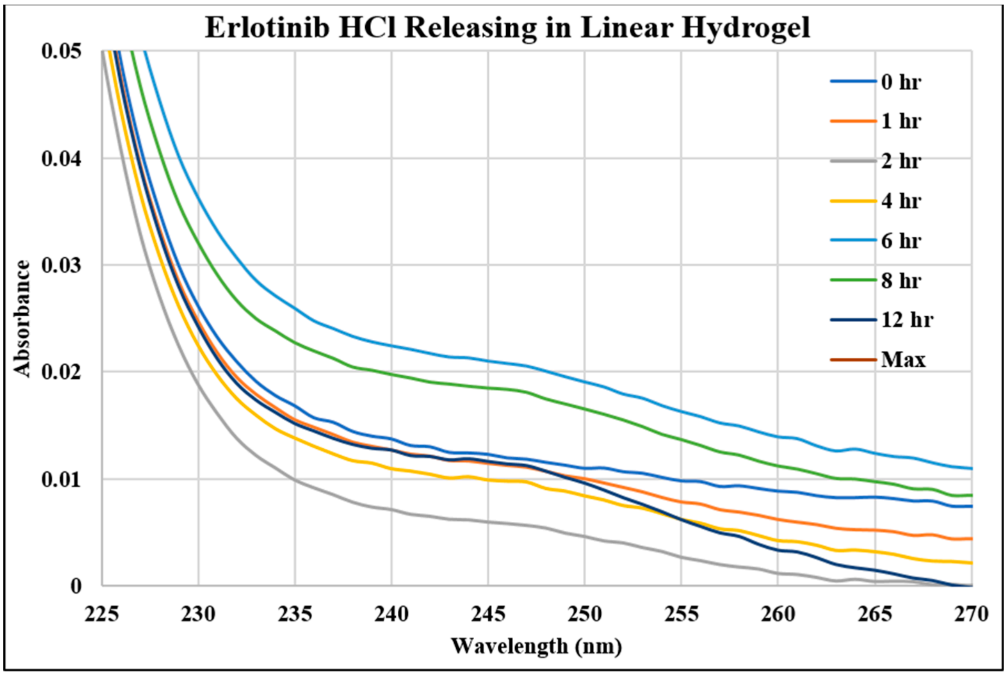

Figure S12: UV-Vis spectra of erlotinib HCl releasing in linear hydrogel.

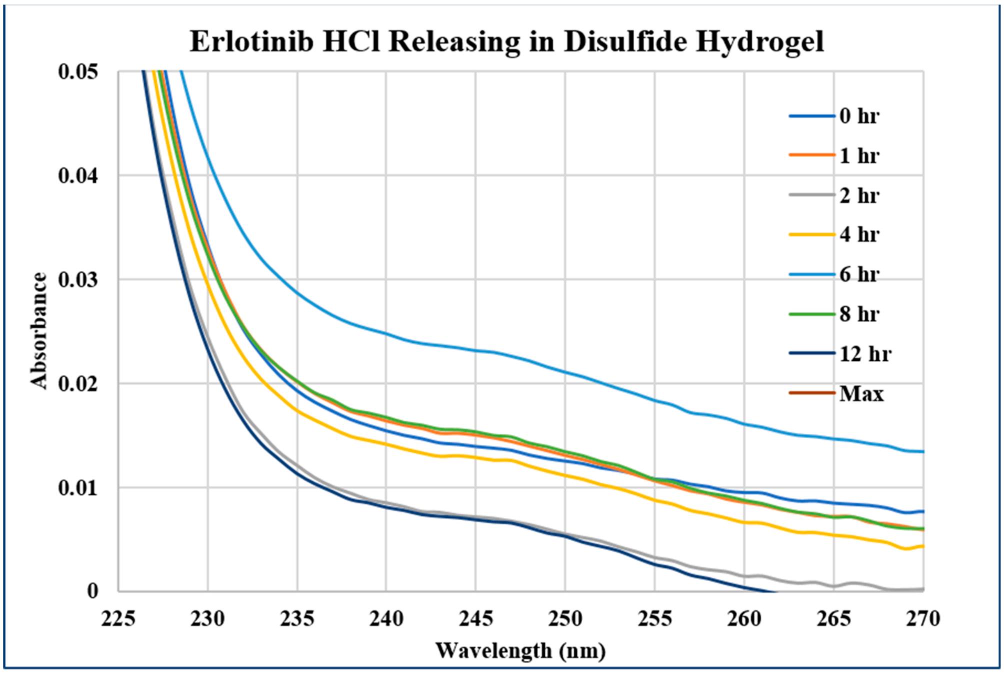

Figure S13: UV-Vis spectra of erlotinib HCl releasing in disulfide crosslinked hydrogel.

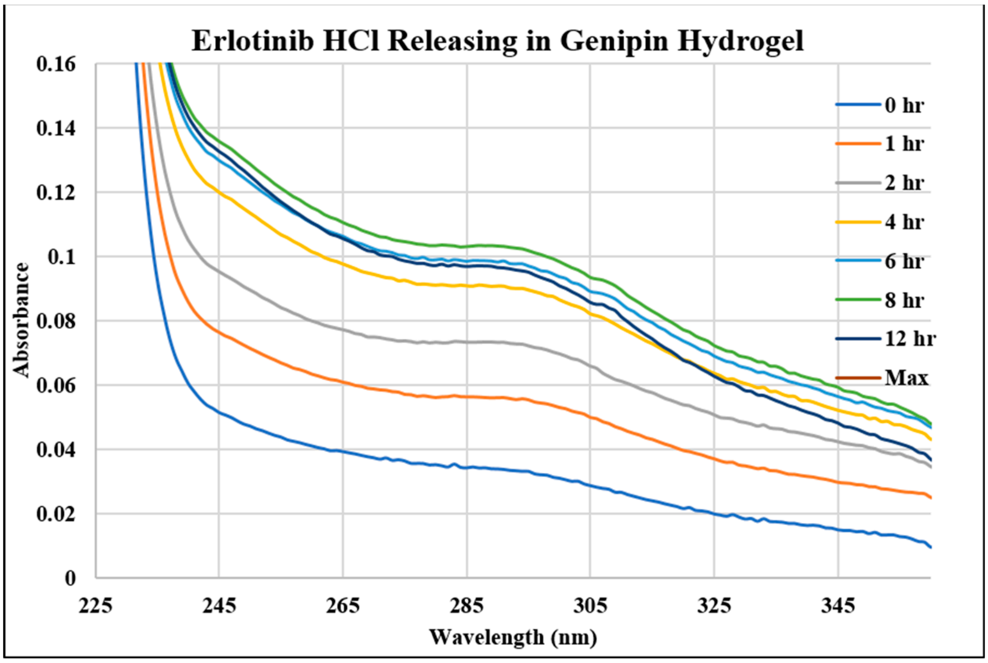

Figure S14: UV-Vis spectra of erlotinib HCl releasing in genipin crosslinked hydrogel.

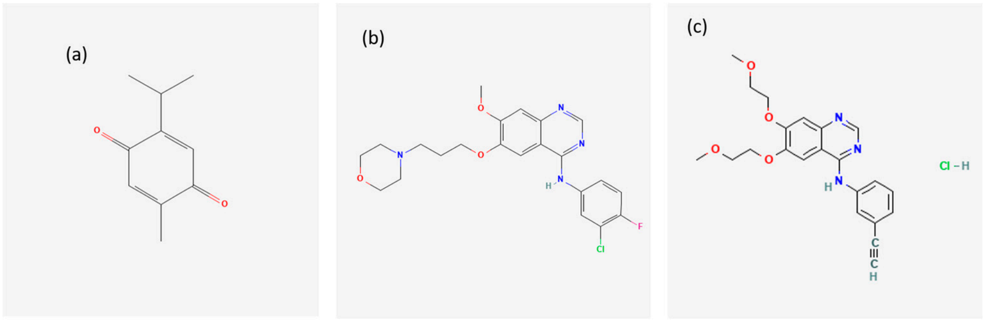

Figure S15: Chemical Structures of three drug molecules used in this study [34,36, and 38].

Table S1: Absorbance while loading

TQ-linear		
Time (hr)	Wavelength (nm)	Absorbance
0	258	0.63
1	258	0.37
2	258	0.19
4	268	0.14
6	268	0.15
8	268	0.14
12	268	0.14

TQ-disulfide		
Time (hr)	Wavelength (nm)	Absorbance
0	248	0.63
1	240	0.37
2	245	1.78477
4	245	2.07139
6	245	1.64693
8	245	1.43
12	248	1.3

TQ-Geipin		
Time (hr)	Wavelength (nm)	Absorbance
0	258	0.66
1	258	0.6
2	258	0.63
4	258	0.6
6	258	0.59
8	258	0.57
12	258	0.58

Gefi-linear		
Time (hr)	Wavelength (nm)	Absorb-ance
0	249	0.381092
1	249	0.425884
2	249	0.407737
4	249	0.399361
6	249	0.403476
8	249	0.394373
12	249	0.385817

Gefi-disulfide		
Time (hr)	Wave-length (nm)	Absorbance
0	249	0.488877
1	249	0.528975
2	249	0.539955
4	249	0.643713
6	249	0.677255
8	249	0.632864
12	249	0.680366

Gefi-Geipin		
Time (hr)	Wavelength (nm)	Absorbance
0	249	0.41
1	249	0.42
2	249	0.39
4	249	0.41
6	250	0.43
8	250	0.44
12	251	0.5

Erlo-linear		
Time (hr)	Wave-length (nm)	Absorbance
0	248	0.657407
1	248	0.612893
2	248	0.603518
4	248	0.561995
6	248	0.551028
8	248	0.541116
12	248	0.483314

Erlo-disulfide		
Time (hr)	Wave-length (nm)	Absorbance
0	248	0.651088
1	248	0.798057
2	248	0.811627
4	248	0.806681
6	248	0.802606
8	248	0.769969
12	248	0.723759

Erlo-Geipin		
Time (hr)	Wavelength (nm)	Absorbance
0	248	0.606586
1	248	0.586611
2	248	0.594968
4	248	0.586776
6	248	0.593798
8	248	0.594478
12	248	0.609028

Table S2: Absorbance while loading

TQ-linear		
Time (hr)	Wavelength (nm)	Absorbance
0	230	0.035237
1	230	0.032887
2	230	0.036008
4	230	0.042045
6	230	0.037243
8	230	0.041569
12	230	0.036613

TQ-disulfide		
Time (hr)	Wavelength (nm)	Absorbance
0	230	0.039494
1	230	0.037922
2	230	0.050545
4	230	0.033968
6	230	0.037581
8	230	0.047094
12	230	0.048826

TQ-Geipin		
Time (hr)	Wavelength (nm)	Absorbance
0	255	0.061211
1	255	0.059402
2	255	0.058166
4	255	0.056652
6	255	0.054477
8	255	0.06433
12	255	0.073729

Gefi-linear		
Time (hr)	Wavelength (nm)	Absorbance
0	249	0.013348
1	249	0.01096
2	249	0.005576
4	249	0.007574
6	249	0.010286
8	249	0.010186
12	249	0.01294

Gefi-di-sulfide		
Time (hr)	Wavelength (nm)	Absorbance
0	249	0.018162
1	249	0.016317
2	249	0.012845
4	249	0.014122
6	249	0.016248
8	249	0.015704
12	249	0.016912

Gefi-Geipin		
Time (hr)	Wavelength (nm)	Absorbance
0	295	0.012584
1	295	0.017304
2	295	0.013637
4	295	0.017855
6	295	0.021833
8	295	0.019792
12	295	0.027235

Erlo-linear		
Time (hr)	Wavelength (nm)	Absorbance
0	247	0.011821
1	247	0.011116
2	247	0.005613
4	247	0.00975
6	247	0.020523
8	247	0.018125
12	247	0.011276

Erlo-disulfide		
Time (hr)	Wavelength (nm)	Absorbance
0	247	0.013496
1	247	0.014353
2	247	0.006685
4	247	0.012518
6	247	0.022537
8	247	0.014873
12	247	0.006557

Erlo-Geipin		
Time (hr)	Wavelength (nm)	Absorbance
0	294	0.033254
1	294	0.055653
2	294	0.072658
4	294	0.090056
6	294	0.097607
8	294	0.10231
12	294	0.095544
